# The Role of Mesenchymal Stem Cells in Treating Diabetic Kidney Disease: Immunomodulatory Effects and Kidney Regeneration

**DOI:** 10.7150/ijms.103806

**Published:** 2025-03-03

**Authors:** Po-Jen Hsiao, Wen-Yi Kao, Li-Chin Sung, Chia-Yi Lin, Liam Li-An Tsou, Yung-Hsi Kao, Chu-Lin Chou, Kung-Ta Lee

**Affiliations:** 1 Division of Nephrology, Department of Internal Medicine, Taoyuan Armed Forces General Hospital, Taoyuan, Taiwan.; 2 Division of Nephrology, Department of Internal Medicine, Tri-Service General Hospital, National Defence Medical Centre, Taipei, Taiwan.; 3 Department of Life Sciences, National Central University, Taoyuan, Taiwan.; 4 Department of Biochemical Science and Technology, National Taiwan University, Taipei, Taiwan.; 5 Division of Cardiology, Department of Internal Medicine, Shuang Ho Hospital, Taipei Medical University, New Taipei City, Taiwan.; 6 Division of Cardiology, Department of Internal Medicine, School of Medicine, College of Medicine, Taipei Medical University, Taipei, Taiwan.; 7 Taipei Medical University-Research Centre of Urology and Kidney, Taipei Medical University, Taipei, Taiwan.; 8 Division of Cardiology, Department of Internal Medicine, Tri-Service General Hospital, Taipei, Taiwan.; 9 Graduate Institute of Clinical Medicine, College of Medicine, National Taiwan University, Taipei, Taiwan.; 10 Nephrology Division, Department of Medicine, Mount Sinai Hospital, New York, NY, USA.; 11 Biochemistry, Department of Chemistry, Hofstra University, Hempstead, New York, USA.; 12 Division of Nephrology, Department of Internal Medicine, School of Medicine, College of Medicine, Taipei Medical University, Taipei, Taiwan.; 13 Division of Nephrology, Department of Internal Medicine, Shuang Ho Hospital, Taipei Medical University, New Taipei City, Taiwan.; 14 Division of Nephrology, Department of Internal Medicine, Hsin Kuo Min Hospital, Taipei Medical University, Taoyuan City, Taiwan.; Po-Jen Hsiao, Wen-Yi Kao, and Li-Chin Sung contributed equally.

**Keywords:** mesenchymal stem cells, diabetic nephropathy, diabetic kidney disease, immunomodulation, immunomodulatory effects, kidney regeneration

## Abstract

**Background:** Diabetic kidney disease (DKD), also known as diabetic nephropathy (DN), is characterized by progressive glomerulosclerosis and chronic inflammation. The potential of mesenchymal stem cells (MSCs) in treating DKD could be explored.

**Methods:** In this study, a streptozotocin (STZ)-induced type 1 diabetes mellitus (T1DM) DKD mouse model was utilized to investigate the renoprotective potential of human umbilical cord-derived mesenchymal stem cells (hUC-MSCs) through immunohistochemical, histopathological, and biochemical analyses. Two separate experiments were conducted to assess the therapeutic efficacy of hUC-MSCs in a DN mouse model. The first experiment determined the optimal dose by assigning the body weight and food intake alterations, serum cytokines and kidney function changes post hUC-MSCs treatment. STZ-induced DKD mice were divided to four groups: DKD control and other three hUC-MSCs treatment groups (low-dose: 3x10^6^, intermediate (middle)-dose: 1x10^7^, and high-dose: 3x10^7^ cells/kg), with intravenous administration at weeks 8, 10, and 12 over 14 weeks. The second experiment evaluated treatment frequency, with mice assigned to hUC-MSCs x1, x2, and x3 groups (3x10^7^ cells/kg) administered at weeks 5, 6, and 7 across 12 weeks, assessing the kidney histology and morphometry changes.

**Results:** In the first experiment, the body weight and food intake showed no significant alterations among the DN and other 3 hUC-MSCs treatment groups. Compared to the DKD control group, only high-dose hUC-MSCs (3x10^7^ cells/kg) treatment group significantly reduced the levels of inflammatory cytokines (IL-1β, and TNF-α) (p <0.05). Additionally, the urine albumin-to-creatinine ratio (UACR) levels in the high-dose hUC-MSCs (3×10⁷ cells/kg) treatment group showed a decreasing trend compared to those in the DN control group (p = 0.06). In the second experiment, the hUC-MSCs x3 treatment group (3×10⁷ cells/kg) significantly alleviated kidney histopathology compared to the DKD group (p <0.05).

**Conclusion:** hUC-MSCs treatment may present a potential avenue for reversing glomerulosclerosis and mitigating inflammation in DKD mice. The long-term therapeutic benefits of MSCs-based treatments in patients with DKD and other kidney diseases could be further investigated.

## Introduction

The increasing incidence of end-stage renal disease (ESRD) is a global phenomenon. Diabetic kidney disease (DKD), also known as diabetic nephropathy (DN), is a severe complication of diabetes mellitus (DM) and the leading cause of ESRD worldwide 1. Compared with patients with other types of diabetes, DKD patients have a higher mortality rate, and most of the deaths are due to cardiovascular events 2. DKD usually manifests as albuminuria, a progressive decline in renal function, and glomerulosclerosis and increases the risk for cardiovascular disease 3-5. Until now, however, specific therapies to restore kidney function or slow the progression of DKD remain unavailable. Despite the present therapeutic options for controlling blood glucose and blood pressure, including angiotensin-converting enzyme inhibitors (ACEIs), angiotensin receptor blockers (ARBs), direct renin inhibitors (DRIs) and sodium-glucose cotransporter-2 (SGLT2) inhibitors, kidney damage and kidney function deterioration still inevitably occur in a majority of patients with DN and chronic kidney disease (CKD), which consequently progresses to ESRD 5-9. Globally, finding a new and safe strategy for treatment is of utmost importance for diabetic patients and for decreasing medical costs.

DKD is triggered by mesangial proliferation and the accumulation of extracellular matrix (ECM) proteins in the mesangial region, resulting in nodular or diffuse glomerulosclerosis 10. Concerning the pathological mechanisms underlying diabetic nephropathy, transforming growth factor-β (TGF-β) has been recognized as a critical regulator cytokine that mediates this pathophysiology 11, 12. The intracellular SMAD pathway transduces TGF-β signals and is responsible for collagen type 1 transcription and integrity 13. Nevertheless, the involvement of other pathways supporting TGF-β/SMAD3 signalling might change the outcome of fibrosis. For example, the PI3K/Akt pathway has been implicated in a crucial pathway in TGF-β-mediated collagen type 1 accumulation 14. Moreover, there is evidence that high glucose levels induce collagen type 1 accumulation in mesangial cells and increase PI3K/Akt activity 15, 16. These findings suggest essential cross-talk between the various pathways resulting in diabetic nephropathy.

Mesenchymal stem cells (MSCs) are used systemically or locally to treat many diseases because they have high potential for self-regeneration and differentiation 17, 18. Stem cells are self-regenerating pluripotent cells that can be classified as embryonic stem cells, adult stem cells, or induced pluripotent stem cells. Among these, adult stem cells can be isolated from umbilical cord blood, bone marrow, fat tissue, and deciduous teeth. MSCs have been used for the treatment of inflammatory diseases 19, tissue regeneration and repair 20, the prevention of transplant rejection 21, and other clinical applications.

The use of MSCs has garnered considerable attention due to the regenerative and immunomodulatory properties of these cells. MSCs are used systemically or locally to treat DN because they have high potential for self-regeneration and differentiation 17, 18. Compared with MSCs from other sources, MSCs collected from the umbilical cord are considered to have greater proliferative and differentiation abilities because of their greater ability to express paracrine factors 22. A previous study reported the successful differentiation of MSCs into insulin-producing cells for diabetes cell therapy 23. In addition, MSCs are considered immunomodulators that modulate key inflammatory cell types and interact with cells involved in innate and adaptive immunity such as T cells, natural killer cells, B cells, and dendritic cells 24. MSCs and cell-secreted extracellular vesicles (EVs) have also been proposed to mediate the downregulation of inflammatory markers, including interleukin-1β (IL-1β), tumor necrosis factor-alpha (TNF-α), and interleukin-6 (IL-6), while increasing the levels of protective cytokines such as interleukin-10 (IL-10), prostaglandin E2, and indoleamine 2,3-dioxygenase 25. Bone degeneration studies have shown that MSCs can decrease the secretion of macrophage inflammatory protein and monocyte chemoattractant protein 26. In rodent models of acute lung injury (ALI), Gupta and coworkers demonstrated that MSCs increase the expression levels of the anti-inflammatory cytokine interleukin-10 27.

Human umbilical cord-derived MSCs (hUC-MSCs) have a more regular doubling time, rarely produce teratomas, and have a greater differentiation capability than bone marrow-derived MSCs. The expression level of major histocompatibility complex classes I and II is reduced in hUC-MSCs, leading to decreased immunogenicity 28. The antifibrotic and immunomodulatory properties of hUC-MSCs have been observed through their effects on prostaglandin E2 29. hUC-MSCs decreased reactive oxygen species levels and inflammatory processes in ALI *in vivo* 30, 31. Additionally, hUC-MSCs alleviated inflammation and augmented the percentage of regulatory T cells in ovalbumin-induced murine models 32. To date, some data indicate that MSCs might alleviate the symptoms of comorbidities of diabetes mellitus (DM) 33-35. However, the potential therapeutic effects of hUC-MSCs on streptozotocin (STZ)-induced type 1 diabetes mellitus (T1DM) DKD remain to be elucidated. The aim of this research was to investigate the effects of hUC-MSCs on inflammation and glomerulosclerosis in DKD mice, with a focused review on animal models and the clinical implications of MSCs-based/EVs therapy.

## Materials and methods

### STZ-induced DKD animal model

Specific pathogen-free (SPF) male C57BL/6 mice (8 weeks old; National Laboratory Animal Centre, Taiwan) were used in this study. The animals were housed at 22 ± 2°C under a 12-h light/12-h dark cycle with free access to food and water. This study was approved by the Institutional Animal Care and Use Committee of Fu Jen Laboratory Animal Centre (Taiwan) (IACUC permission no. P11004) and with the 1964 Helsinki declaration and its later amendments or comparable ethical standards. The protocol for STZ-induced T1DM DKD is briefly described as follows. C57BL/6 mice were intraperitoneally injected with 55 mg/kg/day STZ (Sigma‒Aldrich, St. Louis, MO) for 5 consecutive days 36, 37. After 2-4 weeks, mice with a fasting blood glucose concentration >200 mg/dL were considered diabetic and were used in the DKD experiments. Blood glucose levels were measured using an Accu-Check Performa glucometer (Roche, Basel, Switzerland).

### hUC-MSCs preparation

hUC-MSCs used in this study were obtained from Meridigen Biotech Co., Ltd. (Taipei, Taiwan), and their preparation followed the International Society for Cellular Therapy Guidelines. Briefly, umbilical cord tissue was digested with collagenase and incubated in α-minimal essential culture medium. hUC-MSCs were passaged upon reaching 80-90% confluence up to the sixth generation. For long-term storage, the cells were suspended in CryoStor CS10 and cryopreserved in a vapor-phase liquid nitrogen tank 38. Prior to administration, hUC-MSCs were diluted in clinical-grade normal saline (NS) and 2% clinical-grade human serum albumin (HSA) and intravenously administered via the tail vein.

### hUC-MSCs treatment of STZ-induced DKD animal model

To evaluate the *in vivo* efficacy of hUC-MSCs, 2 different experiments were performed. The first experiment determined the optimal dose by evaluating changes in body weight, food intake, serum cytokines, and kidney function following hUC-MSCs treatment. The animals were grouped as follows: (i) normal group (without STZ; iv NS with 2% HSA); (ii) DKD control group (NS with 2% HSA) and (iii) low-dose hUC-MSCs (3x10^6^ cells/kg) treatment group, (iv) intermediate (middle)-dose hUC-MSCs (1x10^7^ cells/kg) treatment group, and (v) high-dose hUC-MSCs (3x10^7^ cells/kg) treatment group. Two hundred microlitre hUC-MSCs suspensions (from batches RDWP19001U163006060, RDWP19001U163006061, and RDWP19001U163006062) were administered. All animals were injected intravenously through the tail vein 3 times at 2-week intervals.

The second experiment evaluated treatment frequency by assigning mice to hUC-MSCs X1, X2, and X3 groups (3×10⁷ cells/kg), with treatments administered at weeks 5, 6, and 7 over a 12-week period, and assessing changes in kidney histology and morphology. The animals were divided into (i) normal (without STZ; NS with 2% HSA) and diabetic groups, where the diabetic group was further randomly divided into four groups: (ii) DKD group (NS with 2% HSA), (iii) MSC X1 group, (iv) MSC X2 group, and (v) MSC X3 group, which received 3x10^7^ cells/kg of hUC-MSCs (batch RDWP19001U163006058, RDWP 19001U163006057, and RDWP19001U163006056) via 200-µl iv tail vein injection 1, 2, and 3 times, respectively, with a 1-week interval between administrations. Twenty-four hours before sacrifice, the mice were transferred to a metabolic cage for the collection of urine and the measurement of 24-h urine volume. Access to food and water was not limited.

### Serum cytokine/chemokine biomarkers

Serum was collected by centrifugation at 1500 × g for 30 min at 4°C and stored at -20°C until analysis. To determine the presence of systemic inflammation, the serum concentrations of IL-6, IL-10, IL-1β, and TNF-α were measured using a Mouse Cytokine/Chemokine Magnetic Bead Panel (MHSTCMAG-70K-04; Milliplex, St. Louis, MO) on the Luminex-MAGPIX multiplex immunoassay system according to the manufacturer's instructions. The data were analysed using Milliplex Analyst 5.1 software (EMD Millipore, Billerica, MA, United States).

### Biochemical and urine parameter measurement

Whole-blood samples from treated mice were collected by intracardiac puncture and centrifuged at 2000×g for 20 min to separate the serum. The biochemical parameters analysed included serum (blood urea nitrogen [BUN] and creatinine) and urine (albumin and creatinine) levels, which were measured with a Beckman Coulter AU480. The 24-h creatinine clearance rate (CCR) was calculated based on the 24-h urine volume, serum creatinine concentration and urine creatinine concentration. The urine albumin-to-creatinine ratio (UACR) was the ratio of albumin to creatinine.

### Kidney histopathological staining, Masson's trichrome staining and analysis

All mice were anaesthetized, and their kidneys were removed and preserved in 10% formalin. The kidneys were trimmed, embedded in paraffin, sectioned in 4- to 5-μm sections, and stained with haematoxylin-eosin (HE) and Masson's trichrome (MT). Subsequently, the sections were examined microscopically using an optical microscope (Leica DM2700M, USA). Elbe H, *et al.* demonstrated the amelioration of streptozotocin-induced DN by melatonin, quercetin, and resveratrol in rats. In our study, the semiquantitative scoring of renal histopathological lesions was conducted following the previous report 39. Kidney injury was graded as follows: 0, normal; 1, mild injury; 2, moderate injury; and 3, severe injury. Tubular changes, including hydropic degeneration (swelling/vacuolization), desquamation, brush border loss, and peritubular infiltration, were graded as follows, with a maximum score of 12: 0, normal; 1, mild changes; 2, moderate changes; and 3, severe changes. Glomerulosclerotic injury in MT-stained sections was graded as follows: 0, normal; 1, injury in ≤25% of the glomerular area (mild sclerosis); 2, injury in 25-50% of the glomerular area (moderate sclerosis); and 3, injury in ≥75% of the glomerular area (severe sclerosis) 40.

### Statistical analysis

Differences in the frequency distribution (percentage) of categorical variables between groups were evaluated using the chi-square test, and differences in continuous variables (presented as the mean ± standard deviation [SD]) were evaluated using two-sample t tests or one-way analysis of variance with Dunnett's test. All the statistical analyses were performed using the Statistical Analysis Software SPSS 22.0 (SPSS Inc., Chicago, IL, USA).

## Results

### Changes in body weight and food intake

**Figure 1:**
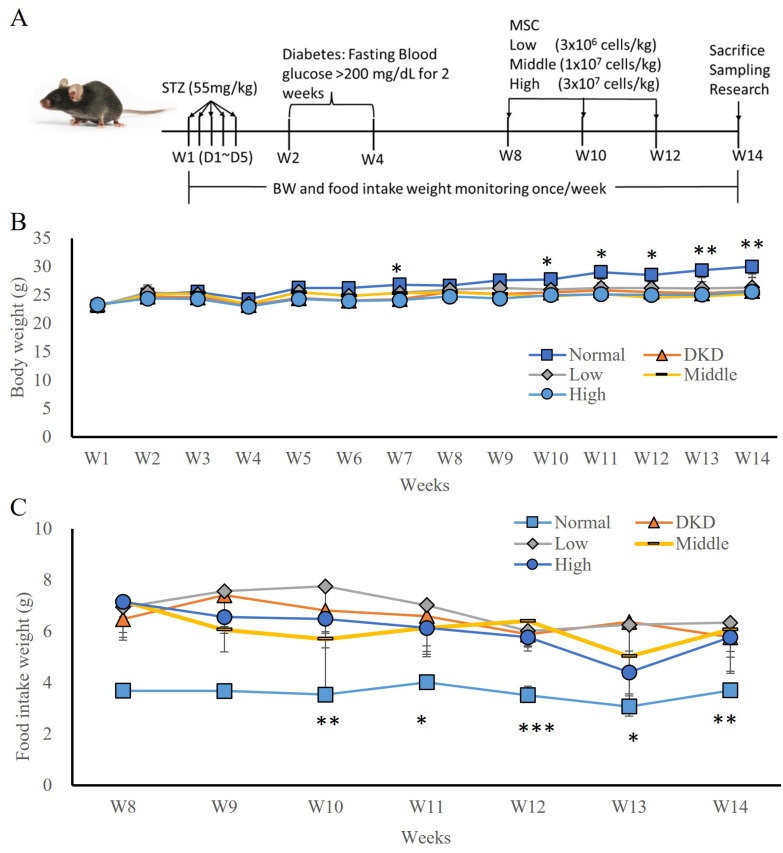
hUC-MSCs treatment STZ-induced DKD animal models and changes in body weight (BW) and food intake following hUM-MSCs treatment. **(A):** Experimental timeline. **(B):** body weight.** (C):** food intake weight. Normal: normal mice; DKD: diabetic mice; Low: diabetic mice treated with MSCs (3 x 10^6^ cells/kg); Middle: diabetic mice treated with MSCs (1 x 10^7^ cells/kg); High: diabetic mice treated with MSCs (3 x 10^7^ cells/kg). Values are expressed as mean ± SD (n = 5~8). * p<0.05; ** p < 0.01; *** p < 0.001 versus DKD group.

In the first experiment, a STZ-induced T1DM hyperglycaemia animal model was established (Fig. 1A) by randomly grouping the animals into 4 groups: DKD, low-dose hUC-MSCs (3x10^6^ cells/kg), intermediate-dose hUC-MSCs (1x10^7^ cells/kg), and high-dose hUC-MSCs (3×10^7^ cells/kg) treatment groups. hUC-MSCs were intravenously administered at weeks 8, 10, and 12 (Fig. 1B). The body weights of the DKD and hUC-MSCs treatment groups were not significantly different, but the body weights of the normal group were significantly different from those of the DKD group at weeks 7, 10, 11, 12, 13, and 14 (Fig. 1B, p <0.05). The weight of food intake during the hUC-MSCs treatment period did not significantly differ between the DKD and hUC-MSCs (low-dose, intermediate-dose, and high-dose) groups, but the food intake of the normal group was significantly different from that of the DKD group (Fig. 1C, p <0.05). Moreover, fasting blood glucose levels did not differ between the DKD and hUC-MSCs groups before or after hUC-MSCs treatment.

### Immunomodulatory effects

**Figure 2:**
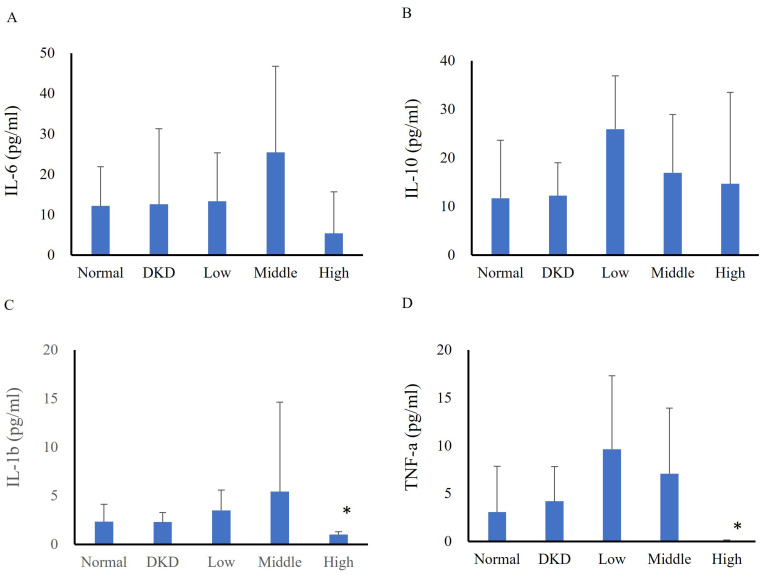
Changes in mouse serum cytokines following hUC-MSCs treatment (N=5-8). **(A)**: IL-6, **(B)**: IL-10, **(C)**: IL-1β, **(D)**: TNF-α. Normal: normal mice; DKD: diabetic mice; Low: diabetic mice treated with hUC-MSCs (3 x 10^6^ cells/kg); Middle: diabetic mice treated with hUC-MSCs (1 x 10^7^ cells/kg); High: diabetic mice treated with hUC-MSCs (3 x 10^7^ cells/kg). Values are expressed as mean ± SD (n = 5~8). * p<0.05 versus DKD group.

In the first experiment, serum cytokine analysis revealed no differences among groups in terms of IL-6 (normal: 12.15±9.73 pg/ml, DKD: 12.56±18.75 pg/ml, low-dose: 13.32±12.01 pg/ml, intermediate-dose: 25.43±21.27 pg/ml, high-dose: 5.34±10.34 pg/ml) (Fig. 2A) or IL-10 levels (normal: 11.70±6.77 pg/ml, DKD: 12.21±6.77 pg/ml, low-dose: 25.90±10.94 pg/ml, intermediate-dose: 16.94±12.01 pg/ml, high-dose: 14.68±18.80 pg/ml) (Fig. 2B). The IL-1β (normal: 2.33±1.81 pg/ml, DKD: 2.30±0.99 pg/ml, low-dose: 3.50±2.07 pg/ml, intermediate-dose: 5.42±9.21 pg/ml, high-dose: 1.00±0.31 pg/ml) (Fig. 2C) and TNF-α (normal: 3.06±4.78 pg/ml, DKD: 4.19±3.64 pg/ml, low-dose: 9.63±7.69 pg/ml, intermediate-dose: 7.08±6.84 pg/ml, high-dose: 0.05±0.09 pg/ml) (Fig. 2D) levels significantly differed between the high-dose group and the DKD group. Changes in serum cytokines of DKD mice following hUM-MSCs treatments were summarized in Table 1.

### Kidney function changes

**Figure 3:**
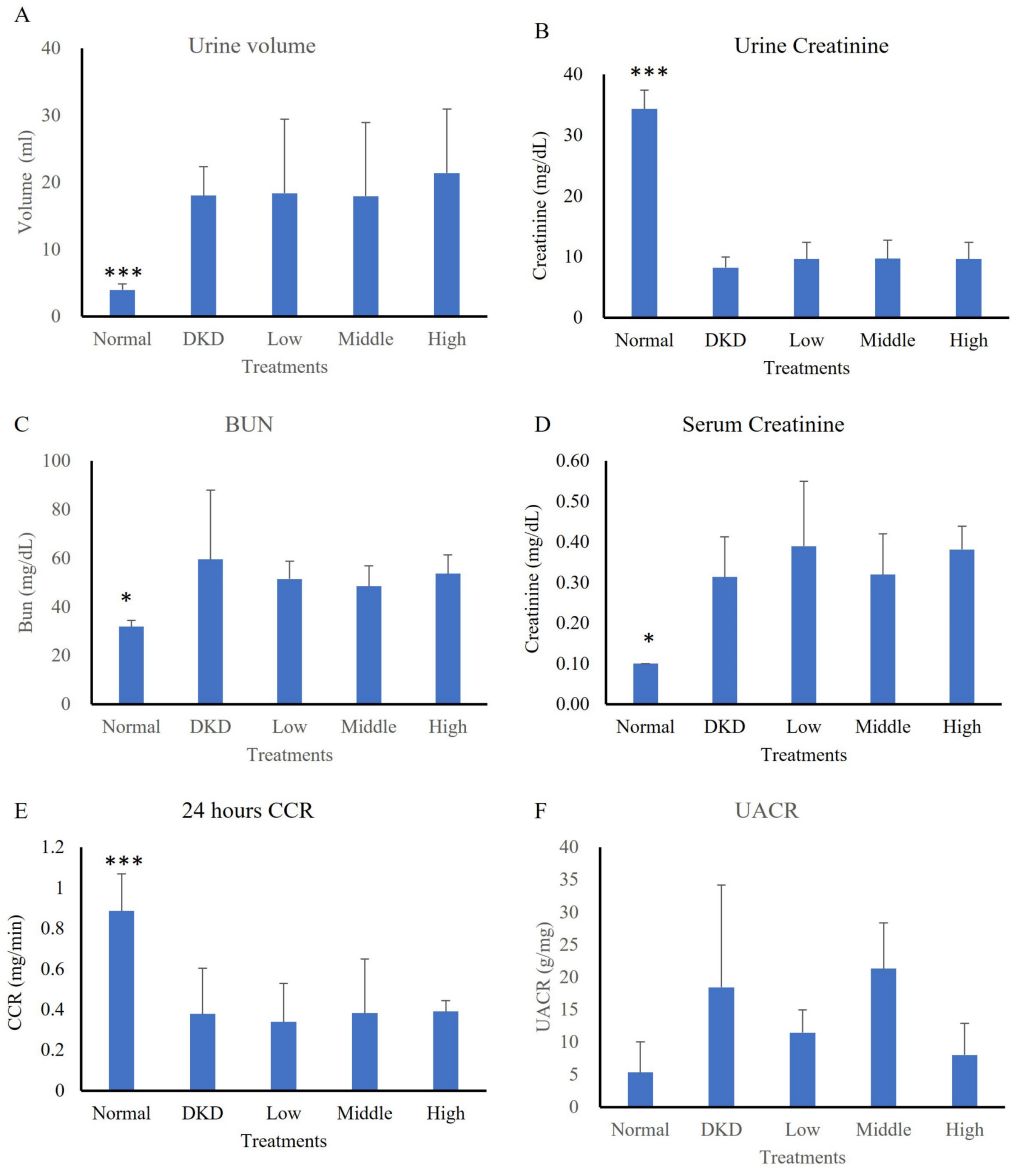
Renal function of streptozotocin-induced DKD animal treated with hUC-MSCs (N=5-8). **(A)**: 24 hours urine volume. **(B)**: Urine creatinine. **(C)**: Blood urea nitrogen (BUN). **(D)**: serum creatinine. **(E)**: 24 hours creatinine clearance rate (CCR). **(F)**: Urine Albumin to Creatinine Ratio (UACR). Normal: normal mice; DKD: diabetic mice; Low: diabetic mice treated with hUC-MSCs (3 x 10^6^ cells/kg); Middle: diabetic mice treated with hUC-MSCs (1 x 10^7^ cells/kg); High: diabetic mice treated with hUM-MSCs (3 x 10^7^ cells/kg). Values are expressed as mean ± SD. * p<0.05; *** p < 0.001 versus DKD group.

In the first experiment model, an analysis of 24-h urine collection, the urine volume of the normal group (3.93±0.89 ml) significantly differed from that of the DKD group (18.04±4.28 ml) (p<0.001), while that of the hUC-MSCs treatment groups was not significantly different (low-dose: 18.37±11.05 ml, intermediate-dose: 17.92±11.00 ml, high-dose: 21.38 ±9.55 ml) (Fig. 3A). The average urine creatinine level of the normal group (34.30±3.07 mg/dL) was also significantly different from that of the DKD group (8.21±1.78 mg/dL) (p<0.001), while those of the hUC-MSCs treatment groups were not significantly different (low-dose: 9.64±2.72 mg/dL, intermediate-dose: 9.68±3.02 mg/dL, high-dose: 9.64±2.72 mg/dL) (Fig. 3B). The average level of albumin in urine did not differ among the groups (normal: 0.18±0.15 mg/dL, DKD: 0.14±0.11 mg/dL, low-dose: 0.11±0.03 mg/dL, intermediate-dose: 0.21±0.11 mg/dL, high-dose: 0.09±0.03 mg/dL) (Fig. 3C). The average serum creatinine level in the normal group (0.10±0.00 mg/dL) significantly differed from that in the DKD group (0.31±0.10 mg/dL) (p<0.05), while the serum creatinine levels in hUC-MSCs treatment groups did not significantly differ (low-dose: 0.39±0.16 mg/dL, intermediate-dose: 0.32±0.10 mg/dL, high-dose: 0.38±0.06 mg/dL) (Fig. 3D). The average 24-h CCR of the normal group (0.89±0.18 ml/min) significantly differed from that of the DKD group (0.38±0.23 ml/min) (p<0.001), while the CCRs of the hUC-MSCs groups did not significantly differ (low-dose: 0.34±0.19 ml/min, intermediate-dose: 0.28±0.27 ml/min, high-dose: 0.39±0.05 ml/min) (Fig. 3E). The UACR levels showed a difference among the 5 groups (normal: 5.33±4.68 mg/g, DKD: 18.45±4.68 mg/g, low-dose: 11.45±3.54 mg/g, intermediate-dose: 21.31±7.06 mg/g, high-dose: 8.01±4.89 mg/g) (Fig. 3F). Results showed a decreasing trend in UACR levels in the high-dose hUC-MSC treatment group compared to the DKD control group (p = 0.063). Changes in kidney function of DKD mice following hUC-MSCs treatments were summarized in Table 2.

### Kidney histopathological evaluations

**Figure 4:**
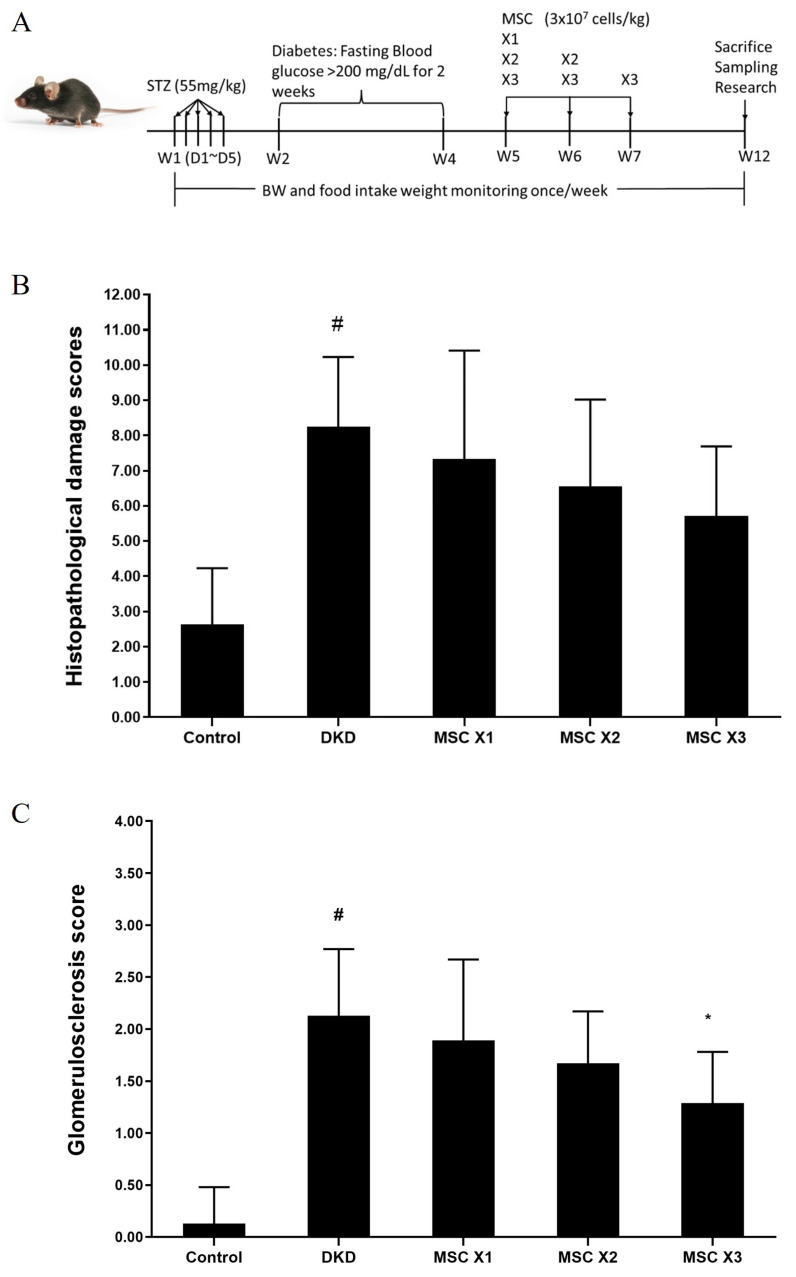
Histopathological changes following hUC-MSCs treatment.** (A):** Experimental timeline. **(B):** Histopathological damage scores DKD group showed significant different with normal group (#: unpaired student's test, p < 0.05). **(C):** Glomerulosclerosis score DKD group showed significant different with normal group (#: unpaired student's test, p < 0.05), in treatment groups, hUC-MSCs (3 x 10^7^ cells/kg) x 3 group showed significant different with DKD group (*: one-way ANOVA, P<0.05). Control: normal mice; DKD: diabetic mice; Values are expressed as mean ± SD (n = 5~8).

**Figure 5:**
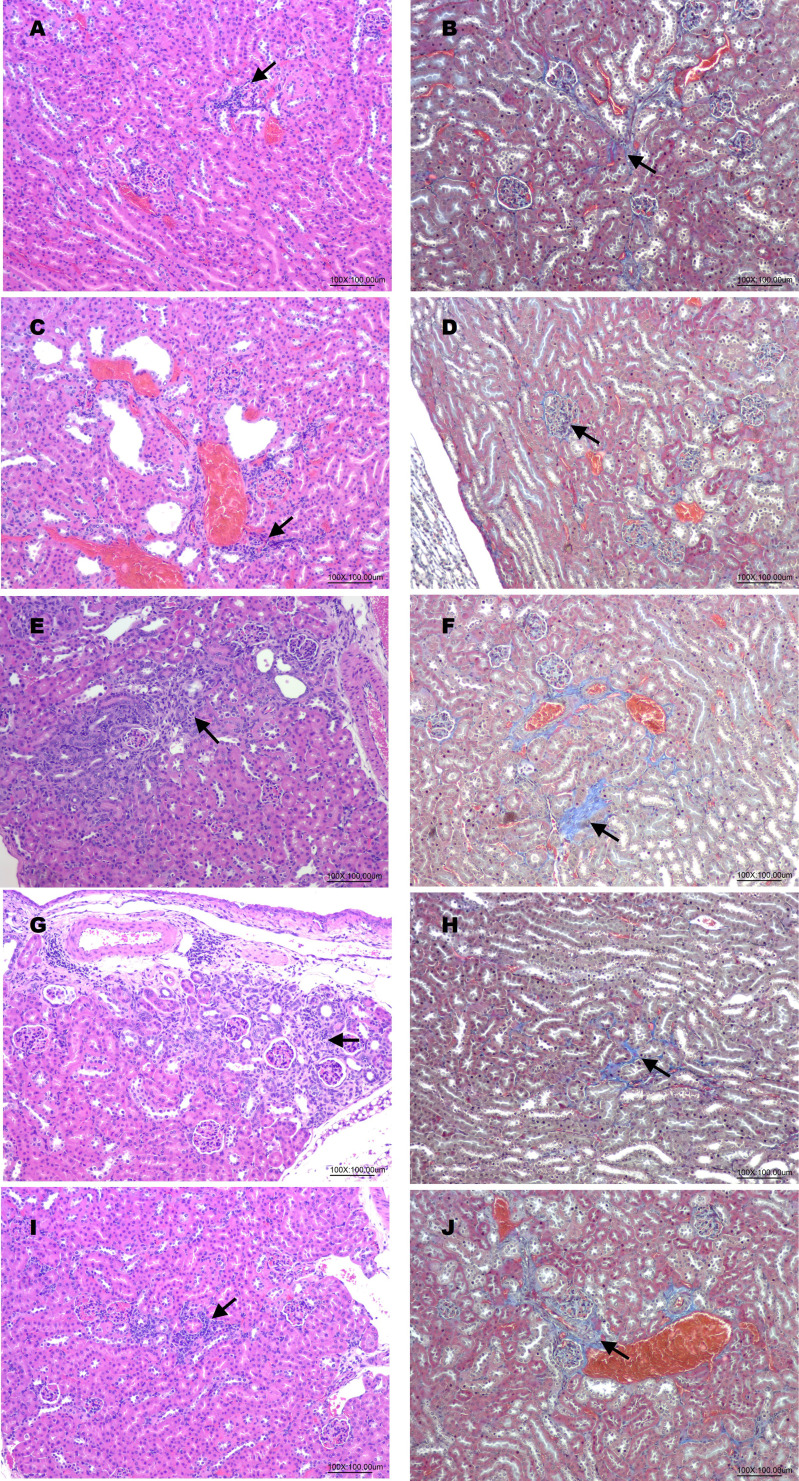
Histopathological changes in haematoxylin-eosin (HE) and Masson's trichrome (MT)-stained kidneys sections in mice. (A, B): Normal (HE, MT); (C, D): DKD (HE, MT); (E, F): hUM-MSCs (3x10^7^ cells/kg) X1 (HE, MT); (G, H): hUM-MSCs (3x10^7^ cells/kg) X2 (HE, MT); (I, J): hUM-MSCs (3x10^7^ cells/kg) X3 (HE, MT). Inflammatory cell infiltrations (arrow) were observed in HE stained sessions. Fibrosis (arrow) was observed in MT-stained sessions.

To determine the potential renal protective effects of hUC-MSCs in treating DKD mice, the second experimental animal model evaluated treatment frequency. Mice were assigned to hUC-MSCs X1, X2, and X3 groups (3×10⁷ cells/kg), with administrations at weeks 5, 6, and 7 over a 12-week period (Fig. 4A). HE staining analysis of kidney tissues indicated inflammation, dilation, degeneration, congestion, basophilia, hyaline casts, and fibrosis. The semiquantitative scores of renal lesions (Table 3) in the normal and DKD groups were significantly different in terms of dilation, degeneration, congestion, and fibrosis. After hUC-MSCs treatment, the congestion scores of the X2 and X3 groups were significantly different from that of the DKD group, and the fibrosis score of the X3 group was also significantly different from that of the DKD group. One-way ANOVA showed that the DKD group was significantly different from the normal group (Fig. 4B). MT staining analysis revealed a significant difference in glomerulosclerosis injury scores between the normal and DKD groups, with all 3 hUC-MSCs treatment groups being significantly different from the DKD group (Table 4, and Fig. 4C). Representative kidneys sections in mice were shown in Fig. 5.

## Discussion

DKD is a major complication of DM and remains a significant challenge in clinical management. Various therapeutic strategies have been explored to slow the progression of DKD, including lifestyle modifications, pharmacological agents, and stem cell-based therapies. Among these approaches, the use of MSCs has garnered considerable attention due to their regenerative and immunomodulatory properties. Previous clinical studies on T1DM and T2DM reported that MSCs derived from human placenta (PL-MSCs), adipose tissue (AD-MSCs), and bone marrow (BM-MSCs) were able to reduce glycated haemoglobin (HbA1c) levels after 3-12 months of treatment 41-45. Our study utilized an STZ-induced DKD mouse model representing T1DM to assess the renoprotective effects of hUC-MSCs through immunohistochemical, histopathological, and biochemical analyses. The results revealed hUC-MSCs treatment in DKD model animals did not significantly affect body weight, food intake, or blood glucose levels in either the DKD or hUC-MSCs treatment groups. However, the normal group showed significant differences in body weight and food intake compared to the DKD and hUC-MSCs treatment groups throughout the experiment, indicating that hUC-MSCs treatment did not have a significant effect on body weight or hyperglycaemia. Lee RH, *et al.* reported that after hUC-MSCs were infused intravenously into mice, 2x10^6^ cells were cleared in 5 min (99±1.07%). Only a few hUC-MSCs differentiated into insulin-producing cells, but these differentiated cells were unable to affect T1DM symptoms 46.

Serum cytokine levels were analysed, and high-dose hUC-MSCs (3x10^7^ cells/kg) treatment significantly decreased IL-1β and TNF-α levels compared to those in the DKD group. Additionally, low-dose hUC-MSCs (3x10^6^ cells/kg) treatment resulted in significant differences in IL-10 levels compared to those in both the DKD and normal groups. These findings suggest that hUC-MSCs treatment may have some impact on inflammatory markers, particularly at higher doses. It has also been reported that UC-MSCs upregulate IL-10 and VEGF expression levels in macrophages through the secretion of PGE2; reduce the levels of proinflammatory factors such as IL-1b, TNF-α, and IL-6 and ameliorate renal interstitial fibrosis in the kidney 43, 47, 48.

In our study, to evaluate the ability of hUC-MSCs to reverse glomerulosclerosis and reduce the effects of proinflammatory factors, 3x10^7^ hUC-MSCs/kg was administered 1, 2, and 3 times, and renal pathology was analysed. We performed HE and MT staining of kidney tissues to assess renal lesions and glomerulosclerosis injury, respectively. The results indicated that the DKD group exhibited inflammation, dilation, degeneration, congestion, basophilia, hyaline casts, and fibrosis. The semiquantitative scores of renal lesions were significantly different between the normal and DKD groups in terms of dilation, degeneration, congestion, and fibrosis. The congestion scores of the X2 and X3 groups were significantly different from those of the DKD group, and the fibrosis scores of the X3 group were also significantly different from those of the DKD group. Li *et al.* 48 reported that a single UC-MSCs treatment ameliorated glomerular abnormalities and interstitial fibrosis in an STZ-induced diabetic mouse model without affecting hyperglycaemia. Notably, He J and Zheng S, *et al.* explored the potential of UC-MSCs in the treatment of diabetic nephropathy 49, 50. Zheng *et al.* also presented that UC-MSCs-derived miR-342-3p inhibited renal tubular epithelial cell pyroptosis by targeting the NLRP3/caspase1 pathway to effectively ameliorate kidney damage and reduce inflammation in a diabetic rat model 50. These results are consistent with the current findings of reduced renal pathology scores following hUC-MSCs treatment, further supporting the potential therapeutic benefit of UC-MSCs in DKD.

### A potential therapeutic role of different types of MSCs and EVs in treating CKD

The effectiveness of cell therapy for CKD depends on the quantity of MSCs reaching the targeted area. In addition, prolonged observation in hUC-MSCs transplantation for systemic lupus erythematosus (SLE) demonstrated the safety of administering a single dose or even a subsequent dose of hUC-MSCs 51-53. Various types of MSCs and EVs are being explored for the treatment of CKD. Table 3 summarizes the utilization of MSCs and EVs from diverse sources in animal models of CKD 54-74. Although BM-MSCs are prevalent in CKD treatment, their purification poses challenges in terms of speed and efficiency. Consequently, alternative options, such as UC-MSCs, PL-MSCs, and AD-MSCs, have emerged as promising alternatives. Compared with BM-MSCs, AD-MSCs offer various advantages, including simplified purification, accelerated proliferation, increased cell viability, easy availability, and stronger immunomodulatory effects. UC-MSCs multiply rapidly and can be mass produced without their efficacy being compromised. PL-MSCs possess a wide range of sources and minimal immunogenicity and are free from ethical concerns. Additionally, compared with BM-MSCs, PL-MSCs may exhibit superior proliferative potential. EVs could be alternatives to MSCs for treating CKD. In treating CKD, MSC-EVs would have the advantages of lower immunogenicity, greater tumorigenicity, and easier management than MSCs. EVs carry complex cargoes of biological molecules, including cytokines, chemokines, growth factors, and nucleic acids. The content of EV cargo is related to the state of the cell of origin and can reflect the phenotype of the releasing cell. Many preclinical animal studies (Table 5) and clinical trials have revealed that MSCs-EVs are effective in treating CKD (Table 6).

### Molecular and biological mechanism of MSCs and EVs

**Figure 6:**
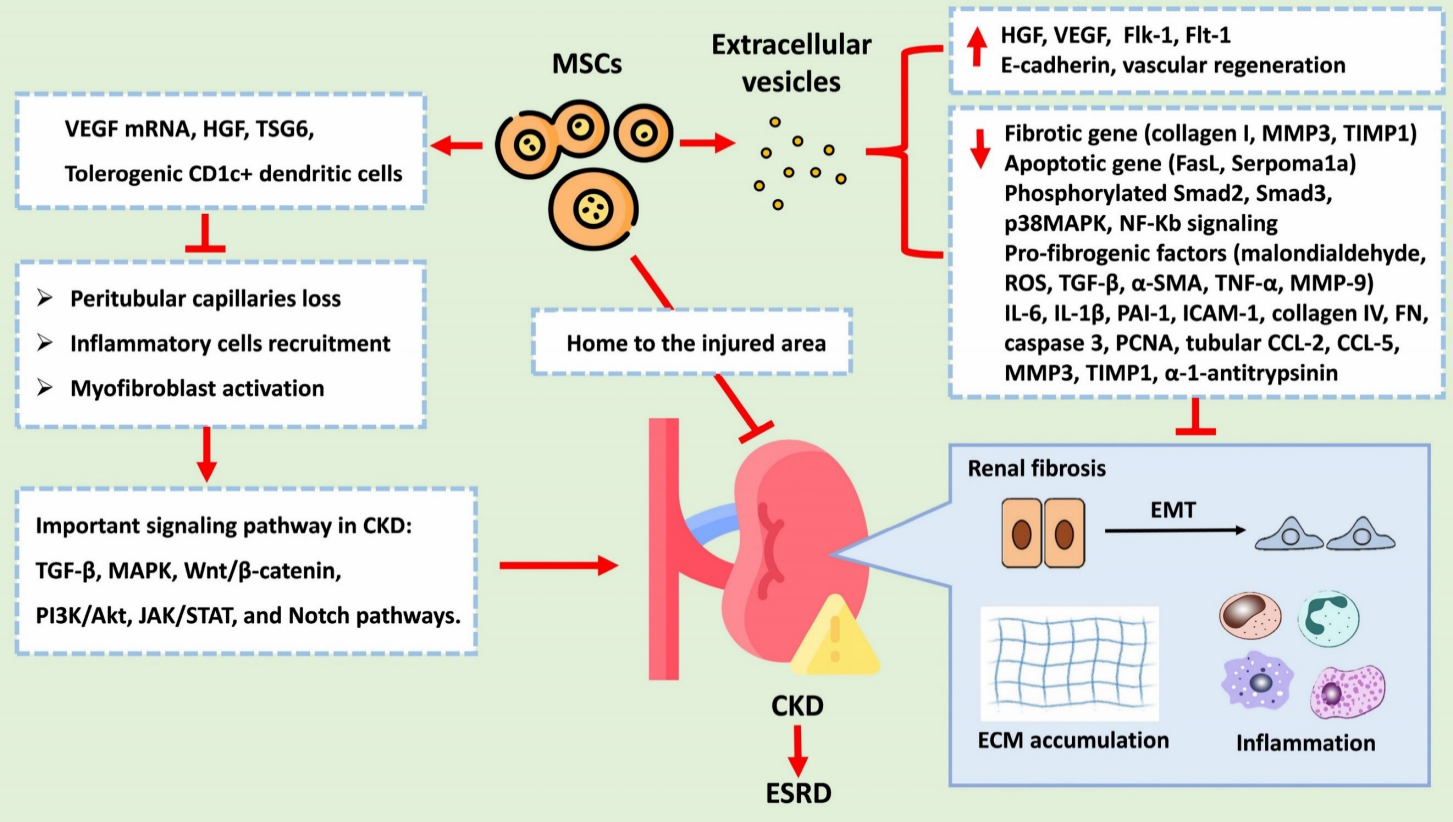
The potential therapeutic role of MSCs and EVs in treating CKD. Abbreviation: VEGF, Vascular Endothelial Growth Factor; HGF, Hepatocyte Growth Factor; TSG6, Tumor Necrosis Factor-Stimulated Gene 6; TGF-B, Transforming Growth Factor Beta (already listed in the previous response); MAPK, Mitogen-Activated Protein Kinases; PI3K/Akt, Phosphoinositide 3-Kinases/Protein Kinase B; JAK/STAT, Janus Kinase/Signal Transducer and Activator of Transcription; Notch, Notch Signaling Pathway. MMP3, Matrix Metallopeptidase 3; TIMP1, Tissue Inhibitor of Metalloproteinases 1; FasL, Fas Ligand; Smad2, Phosphorylated Smad2; Smad3, Phosphorylated Smad3; p38MAPK, Phosphorylated p38 Mitogen-Activated Protein Kinases; NF-Kb, Nuclear Factor Kappa-light-chain-enhancer of activated B cells; ROS, Reactive Oxygen Species; TGF-B, Transforming Growth Factor Beta; a-SMA, Alpha-Smooth Muscle Actin; TNF-a, Tumor Necrosis Factor Alpha; MMP-9, Matrix Metallopeptidase 9; IL-6, Interleukin 6; IL-18, Interleukin 18; PAI-1, Plasminogen Activator Inhibitor-1; ICAM-1, Intercellular Adhesion Molecule 1; PCNA, Proliferating Cell Nuclear Antigen; CCL-2, C-C Motif Chemokine Ligand 2; CCL-5, C-C Motif Chemokine Ligand 5.

CKD is characterized by various pathological changes, with renal fibrosis being a defining feature. Pathological changes in CKD include a loss of peritubular capillaries, the recruitment of inflammatory cells, the activation of myofibroblasts, epithelial-to-mesenchymal transition (EMT), and ECM deposition. These processes are primarily induced and regulated by various signalling networks, such as the TGF-β, MAPK, Wnt/β-catenin, PI3K/Akt, JAK/STAT, and Notch pathways. EMT, which is notably triggered by TGF-β1, involves the transition of epithelial cells into mesenchymal cells and is marked by the overexpression of α-SMA and the loss of E-cadherin. ECM deposition is characterized by the overexpression of fibronectin, collagen I, and collagen IV. Despite multidrug treatment, CKD often progresses to ESRD. MSCs have emerged as a promising therapeutic approach for CKD treatment due to their ability to exert renoprotective effects. MSCs can mitigate CKD progression through various mechanisms: 1. Angiogenesis: MSCs can generate VEGF mRNA and other paracrine factors that promote angiogenesis, preventing the loss of peritubular capillaries. 2. Anti-inflammatory effects: MSCs reduce inflammation by promoting tolerogenic dendritic cells and inhibiting T-cell proliferation and differentiation. 3. Fibrosis reduction: MSCs decrease the levels of profibrotic factors such as IL-6, IL-1β, TNF-α, TGF-β, α-SMA, collagen I, and collagen IV while increasing the levels of antifibrotic factors such as FGFs, HGF, and VEGF, all of which inhibits EMT and reduces renal fibrosis. 4. Cellular protection: MSCs protect renal cells from damage by regulating apoptosis and inhibiting fibrogenic signalling pathways such as the p38 MAPK pathway. 5. EVs: MSC-derived EVs carry as cargo biological molecules that can decrease the expression levels of fibrotic and apoptotic genes, increase E-cadherin expression levels, and promote vascular regeneration. Figure 6 summarizes the potential therapeutic role of MSCs in treating CKD. Due to the therapeutic potential of MSCs, the feasibility of MSC-based clinical trials in various kidney diseases has been proposed 75-81. These findings suggest that the therapeutic effects of hUC-MSCs, particularly at high doses and multiple doses, on DKD might involve multiple pathways beyond glycaemic control. Additionally, initial findings from clinical trials and research have demonstrated a potential from these interventions 77-82. Shimasaki M, *et al.* also indicated that enhancing the resilience of MSCs to stress through preconditioning with dexamethasone or hypoxic conditions may promote accelerated osteogenic differentiation post-transplantation 83.

### Strengths and limitations

Our report offers a concise overview of the potential renoprotective impacts of stem cell-based therapies on both acute and chronic renal dysfunction. In summary, MSCs have shown promise in treating renal fibrosis at various stages by addressing cellular damage, fibrogenic signalling activation, fibrogenic execution, and the destruction of fibrogenic tissue. A recent study also demonstrated that co-administration of a traditional Chinese herb, Alpinate Oxyphyllae Fructus (AOF) and ADMSCs confers renal protection against D-galactose-induced aging by attenuating cellular inflammation, reducing oxidative stress, and inhibiting apoptosis in renal cells 84. The multifaceted mechanisms of MSCs make them a potential therapeutic option for kidney damage. However, further research and clinical trials are necessary to fully understand the therapeutic potential of MSCs and to optimize their application in the management of kidney dysfunction, including CKD and acute kidney injury. In our study, histopathological changes were evaluated in HE-stained and MT-stained kidney sections. However, quantitative morphometry could be more useful for assessing alterations in kidney histology. Recently, this approach has been applied to digitized images of histological preparations (Pannoramic MIDI) stained with hematoxylin and eosin or Masson's trichrome, using the freely available Pannoramic Viewer software 85. Moreover, the manuscript delves into the mechanisms governing the process of kidney regeneration triggered by stem cells.

## Conclusion

In summary, our study results demonstrate that hUC-MSCs may effectively inhibit inflammation, and ameliorate the progression of glomerulosclerosis. These findings provide a basis for the clinical use of hUC-MSCs as a new therapeutic approach for DKD. The results add to the growing body of evidence supporting the potential of MSCs-based therapies in CKD treatment. However, the exact mechanisms of action and optimal dosing regimens remain to be fully elucidated. Given the heterogeneity of DKD and CKD, along with the complex interplay of various cellular and molecular pathways, further investigation is needed to fully understand the therapeutic potential of MSCs and EVs.

## Figures and Tables

**Table 1:** Immunomodulatory effects in serum cytokines following hUC-MSCs treatments.

Cytokines	Group				
(pg/ml)	Normal	DKD	Low	Intermediate	High
IL-6	12.15 ± 9.73	12.56 ± 18.75	13.32 ± 12.01	25.43 ± 21.27	5.34 ± 10.34
IL-10	11.70 ± 6.77	12.21 ± 6.77	25.90 ± 10.94	16.94 ± 12.01	14.68 ± 18.80
IL-1β	2.33 ± 1.81	2.30 ± 0.99	3.50 ± 2.07	5.42 ± 9.21	1.00 ± 0.31*
TNF-α	3.06 ± 4.78	4.19 ± 3.64	9.63 ± 7.69	7.08 ± 6.84	0.05 ± 0.09*

Abbreviations: Normal: normal mice; DKD: diabetic mice; Low: diabetic mice treated with hUC-MSCs (3 x 10^6^ cells/kg); Intermediate: diabetic mice treated with hUC-MSCs (1 x 10^7^ cells/kg); High: diabetic mice treated with hUC-MSCs (3 x 10^7^ cells/kg); IL-6: interleukin-6; IL-10: interleukin-10; IL-1β: interleukin-1β; TNF-α: tumor necrosis factor-alpha. Values are expressed as mean ± SD (n = 5~8). * p<0.05 versus DKD group.

**Table 2:** Kidney function changes following hUC-MSCs treatment.

Biochemistry	Group				
	Normal	DKD	Low	Intermediate	High
Urine volume (ml)	3.93 ± 0.89*	18.04 ± 4.28	18.37 ± 11.05	17.92 ± 11.00	21.38 ± 9.55
Urine creatinine (mg/dL)	34.30 ± 3.07*	8.21 ± 1.78	9.64 ± 2.72	9.68 ± 3.02	9.64 ± 2.72
Albuminuria (mg/dL)	0.18 ± 0.15	0.14 ± 0.11	0.11 ± 0.03	0.21 ± 0.11	0.09 ± 0.03
Serum creatinine (mg/dL)	0.10 ± 0.00*	0.31 ± 0.10	0.39 ± 0.16	0.32 ± 0.10	0.38 ± 0.06

Abbreviations: Normal: normal mice; DKD: diabetic mice; Low: diabetic mice treated with hUC-MSCs (3 x 10^6^ cells/kg); Intermediate: diabetic mice treated with hUC-MSCs (1 x 10^7^ cells/kg); High: diabetic mice treated with hUC-MSCs (3 x 10^7^ cells/kg). Values are expressed as mean ± SD (n = 5~8). * p<0.05 versus DKD group.

**Table 3:** Histopathological damage as indicated by semiquantitative scores of renal lesions.

Measurement^a^	Group				
	Normal	DKD	MSCs X1	MSCs X2	MSCs X3
Inflammation	0.63 ± 0.52	1.00 ± 0.00	0.89 ± 0.60	0.56 ± 0.53	0.43 ± 0.53
Dilation	0.25 ± 0.46	1.75 ± 0.71^#^	1.33 ± 1.22	1.78 ± 0.67	2.00 ± 1.00
Degeneration	0.50 ± 0.76	1.63 ± 0.52^#^	1.67 ± 0.71	1.78 ± 0.67	1.43 ± 0.98
Congestion	0.13 ± 0.35	1.25 ± 0.46^#^	0.67 ± 0.50	0.44 ± 0.53^*^	0.57 ± 0.53^*^
Basophilia	0.63 ± 0.52	1.00 ± 0.00	1.33 ± 0.50	0.89 ± 0.60	0.86 ± 0.38
Hyaline casts	0.13 ± 0.35	0.25 ± 0.71	0.11 ± 0.33	0.00 ± 0.00	0.00 ± 0.00
Fibrosis	0.38 ± 0.52	1.38 ± 0.52^#^	1.33 ± 0.50	1.11 ± 0.60	0.43 ± 0.53^*^
Total scores	2.63 ± 1.60	8.25 ± 1.98^#^	7.33 ± 3.08	6.56 ± 2.46	5.71 ± 1.98

Abbreviations: Normal: normal mice; DKD: diabetic mice; MSCs: diabetic mice treated with hUC-MSCs (3 x 10^7^ cells/kg). ANOVA: analysis of variance; SD: standard deviation. ^a^ Values represent the mean ± SD. ^#^ p < 0.05, DKD (Group 2) vs. Control (Group 1); unpaired Student's t test. ^*^ p < 0.05, treated vs. DKD (Group 2); one-way ANOVA followed by Dunnett's test.

**Table 4:** Semiquantitative scores of glomerulosclerotic injury.

Measurement^a^	Group				
	Normal	DKD	MSCs X1	MSCs X2	MSCs X3
Glomerulosclerosis score	0.13 ± 0.35	2.13 ± 0.64^#^	1.89 ± 0.78	1.67 ± 0.50	1.29 ± 0.49^*^

Abbreviations: Normal: normal mice; DKD: diabetic mice; MSCs: diabetic mice treated with hUC-MSCs (3 x 10^7^ cells/kg). ANOVA: analysis of variance; SD: standard deviation. ^a^ Values represent the mean ± SD. ^#^ p < 0.05, DKD group (Group 2) vs. control group (Group 1); unpaired Student's t test. ^*^ p < 0.05, treated vs. DKD (Group 2); one-way ANOVA followed by Dunnett's test.

**Table 5:** Samples of preclinical studies assessing the therapeutic efficacy of MSCs in models of CKD with different aetiologies.

Aetiology	MSC number and source	Route	Main outcomes	Ref
**Diabetes mellitus**				
STZ-induced diabetic mice	0.5 × 10^6^ mice BM-MSCs	Tail vein injection	↓ Blood glucose levels↑ β-pancreatic islet regeneration, prevented renal damage	54
STZ-induced diabetic mice	2.5 × 10^6^ human BM-MSCs	Left cardiac ventricle	↑ Insulin secretion and perhaps ameliorating the renal lesions	55
STZ-induced diabetic mice	EVs from bone marrow or liver	IV	↓ Collagen I, MMP3, TIMP1, FasL, serpina1a, SNAI1, CCL3, BUN, creatinine, fibrosis, EMT, recruitment of macrophages, T cells	56
STZ-induced diabetic rats	2 × 10^6^ UC-MSCs	Tail vein injection	↓ Proteinuria, Scr, BUN, IL-6, IL-1β, TNF-α and TGF-β↑ FGFs, HGF, and VEGF	57
STZ-induced diabetic rats	3 × 10^6^ rat AD-MSCs	IV	Attenuated CKD↓ IL-6, IL-1β, TNF-α, iNOS(+) M1 macrophages↑ IL-10, CD163(+) M2 macrophages	40
**Obstructive nephropathy**				
UUO mice	5 × 10^5^ human BM-MSCs	Tail vein injection	↓ CD68-positive macrophage, PTC loss, renal tubulointerstitial injury and fibrosis↑ Ki67, α-SMA	58
UUO mice	BM-MSC-EVs	IV	↓ Fibrosis, collagen, MMP-9, α-SMA, TGF-βR1	59
UUO mice	1 × 10^6^ human BM-MSCs	IV	↓ InflammationNo antifibrotic effect	60
UUO rats	UC-MSC-CM	Left renal artery	↓ MDA, ROS, expression of TGF-β1, α-SMA, TNF-α and collagen-I, RTE apoptosis	61
UUO rat	UC-MSC-EVs	left renal artery	↓ Apoptosis of NRK-52 E cells, Scr, BUN, oxidative stress, renal tubular injury and tubulointerstitial fibrosis	62
**Hypertension**				
2K1C-induced renovascular hypertension	2 × 10^5^ rat BM-MSCs	IV	Improved renal morphology and microvascular rarefaction; reduced fibrosis, proteinuria and inflammatory cytokines; suppressed intrarenal RAS	63
2K1C induced renovascular hypertension	1 × 10^6^ rat BM-MSCs	IV	↓ Inflammation and oxidative stress↓ Morphological and ultrastructural abnormalities↓ Serum urea and creatinine	64
5/6 NPX rats	0.5 × 10^6^ AD-MSCs	IV	↓Plasma creatinine, damage markers ED-1 and α-SMA↑Pax-2, BMP-7, and VEGF, Oct-4	65
5/6 NPX rats	2 × 10^5^ rat BM-MSCs	Subcapsular injection	↓ SBP↑ Renal function (↓ Albuminuria, Scr)	66
5/6 NPX rats	2 × 10^5^ rat BM-MSCs	IV	↓ Fibrosis indices (collagen I, vimentin, TGF-β, α-SMA), Inflammation	67
1K/DOCA/salt	1 × 10^6^ human BM-MSCs	IV	↓ SBP, inflammation, fibrosis↑ Renal function and morphology	68
High-salt diet (8% NaCl)	5 × 10^6^ rat BM-MSCs	Intrarenal infusion	↓ SBP, inflammasome activation, hypertensive kidney damage	69
MRL/lpr mice (Lupus nephritis)	1 × 10^6^ PD-MSCs	IV	↓NF-κB mRNA levels, phosphor-NF-κB p65, TNF-α, PAI-1, and ICAM-1 expression	70
				
Cisplatin-induced CKD	3 × 10^6^ rat BM-MSCs	IV	↓ Creatinine and urea↓ Inflammation and fibrosis↑ Hepatocyte growth factor	71
**Nephrotoxicity**				
Mouse model of AA induced nephropathy	BM-MSCs-EVs	IV	↓α-SMA, Col1a1, profibrotic gene expression, blood creatinine and BUN, tubular necrosis, interstitial fibrosis, infiltration of CD45 positive immune cells, fibroblasts, and pericytes	72
CsA nephrotoxicity mouse model	BM-MSCs and EVs	Intraperitoneal injection	Improvement in renal outcomesEVs induced a partial recovery	73
**CKD**				
CKD cats	2 × 10^6^ cat AD-MSCs/kg	IV	No adverse effectsNo significant treatment effect on renal function in the 6 weeks after MSC treatment	74

Abbreviations: 2K-1C: 2 kidney, 1 clip model; 5/6 NPX: nephrectomy; AAN: aristolochic acid nephropathy; AD-MSCs: adipose-derived mesenchymal stem cells; BM-MSCs: bone marrow mesenchymal stem cells; BMP-7: bone morphogenetic protein 7; BUN: blood urea nitrogen; CCR: creatinine clearance rate; CKD: chronic kidney disease; CsA: cyclosporine; FasL: Fas ligand; FGFs: fibroblast growth factor; HGF: hepatocyte growth factor; HIF-1α: hypoxia induction factor-1α; IL-10: interleukin 10; IL-1β: interleukin-1β; IL-6: interleukin 6; IV: intravenous; iNOS: inducible nitric oxide synthase; MDA: malondialdehyde; MSCs: mesenchymal stem cells; MVs: microvesicles; PAI-1: plasminogen activator-1; PL-MSCs: placenta-derived mesenchymal stem cells; PTC: peritubular capillary; RAS: renal artery stenosis; ROS: reactive oxygen species; SCr: serum creatinine; SLE: systemic lupus erythematosus; STZ: streptozotocin; TGF-β1: transforming growth factor-β1; TNF-α: tumour necrosis factor alpha; UC-MSC-Exo: exosomes from umbilical cord mesenchymal stem cells; UC-MSCs: umbilical cord blood mesenchymal stem cells; UUO: unilateral ureteral obstruction; VEGF: vascular endothelial-derived growth factor; α-SMA: α-smooth muscle actin.

**Table 6:** Summary of clinical trials on MSCs in the treatment of CKD.

ClinicalTrials.gov identifier	Study type	Conditions	Interventions	Treatment effect
NCT01843387 75	A multicentre, randomized, double-blind, dose-escalating, sequential, placebo-controlled trial	Type 2 DM/advanced DN	BM-MSCs	No acute adverse events
NCT01539902 76	A randomized double-blind, placebo-controlled trial	WHO class III or IV LN	UC-MSCs	MSC infusion had no apparent additional effect over and beyond that of standard immunosuppression
NCT01741857 53	A long-term follow-up predesigned open-label phase II clinical trial	Severe and drug-refractory SLE	BM-MSCs UC-MSCs	Allogeneic MSC transplantation was safe and resulted in long-term clinical remission in SLE patients
NCT00698191 77	A pilot clinical study	Refractory SLE	BM-MSCs	MSC transplantation appeared beneficial in the treatment of patients with SLE refractory to conventional treatment options
NCT02166489 78	A single-arm phase I clinical trial	CKD due to polycystic kidney disease	BM-MSCs	An intravenous infusion of autologous MSCs was safe and well tolerated in ADPKD patients
NCT01741857 79	A pilot clinical study	Refractory SLE	UC-MSCs	UC-MSCs might suppress inflammation in lupus by upregulating tolerogenic DCs
NCT03174587 80	A phase I clinical trial	SLE with active renal disease	BM-MSCs	BM-MSCs were safe and tolerable
NCT02266394 81	A phase 1a escalating dose clinical trial	Atherosclerotic renovascular disease	AD-MSCs	An increase in cortical and whole kidney blood flows in the poststenotic kidney

Abbreviations: ADPKD: autosomal dominant polycystic kidney disease; AD-MSCs: adipose tissue mesenchymal stem cells; BM-MSCs: bone marrow mesenchymal stem cells; DM: diabetes mellitus; DN: diabetic nephropathy; LN: lupus nephritis; SLE: systemic lupus erythematosus; UC-MSCs: umbilical cord blood mesenchymal stem cells; WHO: World Health Organization.
